# Automated preparation of biological samples prior to high pressure liquid chromatography: Part II- The combined use of dialysis and trace enrichment for analysing biological material

**DOI:** 10.1155/S1463924685000402

**Published:** 1985

**Authors:** J. D. H. Cooper, D. C. Turnell

**Affiliations:** Department of Biochemistry Coventry and Warwickshire Hospital Stoney Stanton Road Coventry CVI4FH UK


Journal of Automatic Chemistry/*Journal of Clinical Laboratory Automation*, Vol. 7, No. 4 (October–December 1985), pp. 181–184

### Automated preparation of biological samples prior to high pressure liquid chromatography: Part II – The combined use of dialysis and trace enrichment for analysing biological material

**J. D. H. Cooper and D. C. Turnell**

*Department of Biochemistry, Coventry and Warwickshire Hospital, Stoney Stanton Road, Coventry CV1 4FH, UK*

### Introduction

In the first Part of this article (see p. 177) an automated procedure for the analysis of amino-acids in human physiological fluids, using pre-column derivatization with o-phthalaldehyde/2-mercaptoethanol (OPA/MCE) and high pressure liquid chromatography (HPLC), was described. This method utilizes dialysis to remove high molecular weight components that can poison chromatography columns, and permits both complex and concentrated biological samples to be sampled directly. The intense fluorescence of OPA/MCE derivatives of amino-acids compensates for the poor efficiency of dialysis but limits the application of this sample preparation technique to assays using sensitive detection or those where analytes are present at a high concentration. Trace enrichment has been used previously for concentrating samples [1] and the incorporation of such a device in the dialyser recipient stream should theoretically overcome this limitation and increase the range of HPLC methods to which deproteinization by dialysis may be applied.

This paper reports on the development of an automated sample preparation system incorporating dialysis and trace enrichment for the treatment of biological fluids prior to HPLC [2]. An examination of the system's performance is described using the analysis of therapeutic concentrations of a drug, phenobarbitone, in human serum.

### Materials and methods

### *Instrumentation*

An isocratic HPLC system was used. This consisted of a Gilson 302 pump and a Gilson Holochrome UV detector (Gilson International, Villiers-le-Bel, France) set at a wavelength of 225 nm. The loop on the Rheodyne 7010 valve was replaced by a specially designed traced enrichment cartridge (TEC) (see figure 1), described below. The analytical column used was a 100 mm × 4·6 mm i.d. column packed with Zorbax ODS (Du Pont Ltd, Hitchin, UK); for some experiments this was fitted with a guard column packed with CO : PELL ODS of 25–37 μm diameter (Whatman Ltd, Maidstone, UK).

### *Dialysis and TEC system*

The automated sampling system comprised a modified auto-analyser (AA) sampler (as described in Part I [p. 177], but fitted with an ordinary sample probe), a 6 in dialyser block and cuprophan membrane, and an AA1 peristaltic pump (all from Technicon, Basingstoke, UK). A Gilson 302 HPLC pump fitted with a 5 ml liquid head was included to pump the dialyser recipient stream through the TEC on the Rheodyne valve. This pump was modified by placing a 5V miniature relay across the flow control switch to allow automatic switching. All the timed operations of these device were controlled by a SP4100 integrator (Spectra Physics Ltd, St. Albans, UK) via the external event switches and relays, see Part I. The design of the TEC is shown in figure 1. Hypersil 5 μm ODS (Shandon Southern Products Ltd, Runcorn, UK), was loaded into the retaining washer. This was carried out by pipetting a 1 ml slurry of Hypersil (20 mg/ml in acetone) into a 4·6 mm o.d. polypropylene tube compressed onto the washer and applying a vacuum to the outlet of the cartridge.

### Reagents

The drugs phenobarbitone and hexobarbitone were obtained from Sigma London Chemical Company Ltd, Poole, UK. Acetonitrile Far UV grade from Fisons Scientific Apparatus, Loughborough, UK. Unless otherwise stated, all chemicals were analytical grade and were supplied by BDH Chemicals Ltd, Poole, UK. Water purified through activated carbon and an ion exchange resin (Spectrum C system, Elga Ltd, High Wycombe, UK) was used for all reagent preparations.

### *Stock phenobarbitone solution*

600 mg of phenobarbitone was dissolved in 100 ml methanol.

### *Internal standard solution*

1000 mg of hexobarbitone was dissolved in 100 ml of methanol.

*Elution solvent*

The HPLC solvents were filtered through a 0·45 μm pore size filter (Anachem Ltd, Luton, UK) and degassed with helium before use. The elution solvent was acetonitrile : water (30 : 70 by volume) at a flow rate of 2·0 ml/min.

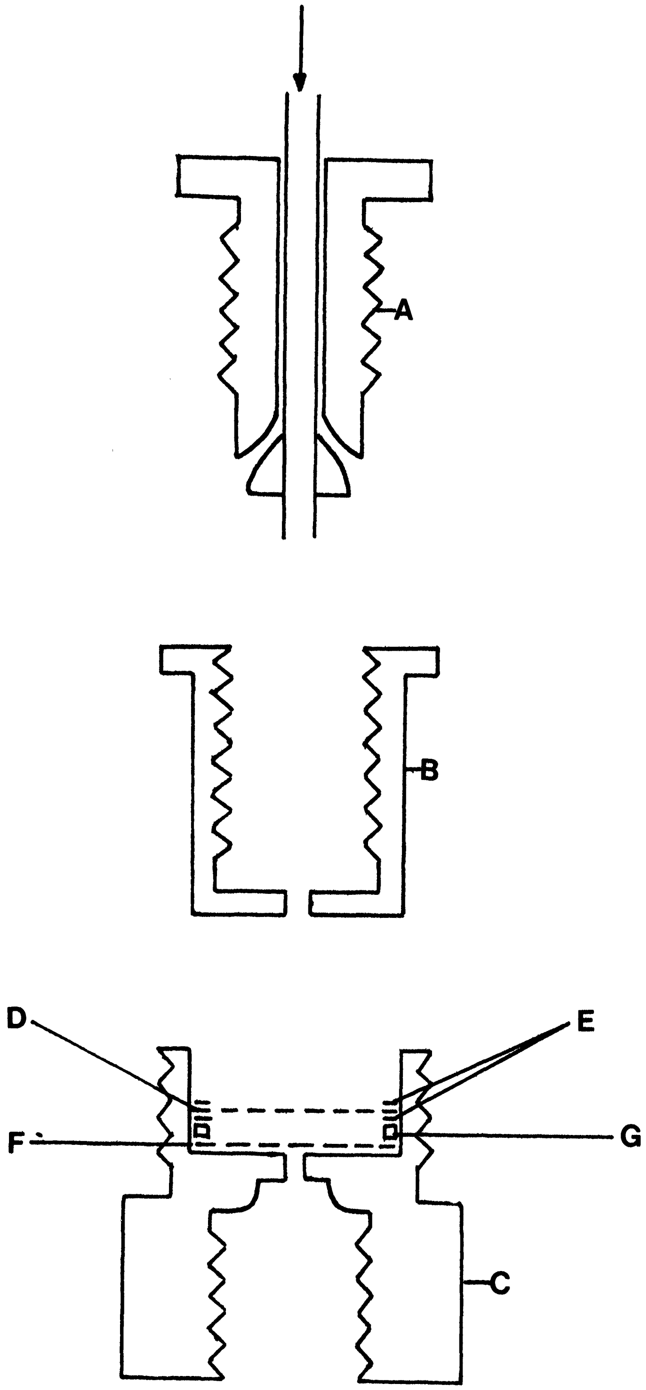

*Figure 1. Sectional view of the TEC; the arrow indicates direction of solvent flow; A, half of a 1/16 in union with its centre hexagonal 5/16 in flats reduced in size; B, machined 1/16 in nut; C, inverted female end fitting, reduced in depth; D, 35 μm top mesh (1/4 in); E, teflon washers; F, 1 μm bottom mesh (1/4 in); G, retaining washer for trace enrichment material. The flats remaining on A pass through the 1/4 in hole in the 1/4 in nut, which, when tightened, bear on the remaining 5/16 in flats on B.*

### Procedures

*Dialyser and TEC system*

The configuration of the system is shown in figure 2. The timing diagram for treating one sample operation is shown in figure 3; the system operates as follows: serum specimens are loaded into sample cups and placed on the sampler, the integrator activated the sampler to introduce the probe into the sample. The AA pump is energized and the sample is aspirated into the dialyser block until the donor channel is completely filled. The sample pump is switched off and the probe returned to the wash position on the sampler. The Gilson 302 pump is energized and water is aspirated through the recipient side of the dialyser and into the TEC. The injection valve is then switched to the inject position. The chromatography solvent elutes the analytes from the TEC onto the analytical column and the integration of the chromatography is initiated. The sample pump is energized, and, with the TEC pump still running, the system is washed out with water. The injection valve is switched to the load position and the TEC re-equilibrated with water via the TEC pump. After re-equilibration of the TEC, both pumps are switched off. At the end of the chromatography and report, the cycle is repeated.

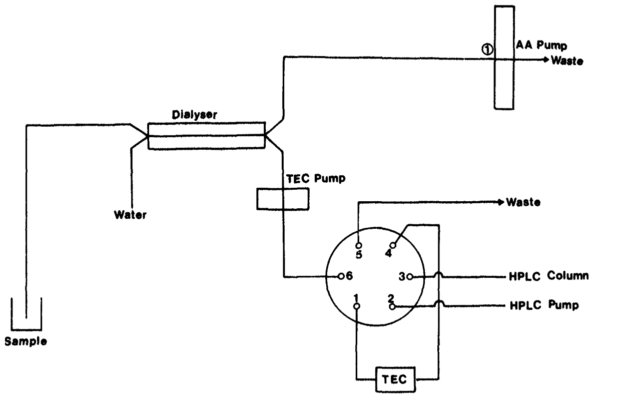

*Figure 2. Schematic diagram of the dialysis/TEC system. Flow rate of pump tube 1 = 0·80 ml/min.*

*Quantitation*

The phenobarbitone peak was identified by its relative retention time to the reference peak and the peak areas determined by the SP4100 integrator.

### System design and validation

*TEC design*

A diagram of the TEC is shown in figure 1. By using different thickness stainless-steel washers, the amount of hypersil packed into the cartridge can be varied. Ideally, the minimum quantity of packing material is required to minimize the pressure drop across the TEC and to reduce the re-equilibration time of the TEC with water after its exposure to the chromatographic solvent. However, the volume of aqueous dialysate passed through the TEC is dependent on the TEC capacity (K′) for the analyte. This

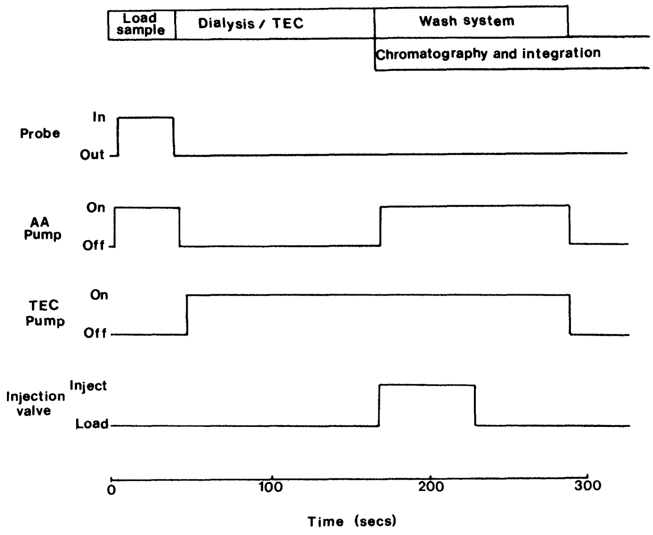

*Figure 3. Timing diagram of the sample preparation system.*

was experimentally determined by replacing the analytical HPLC column with the TEC and injecting a concentrated solution of phenobarbitone in water via the injection valve fitted with a 20 µl loop. Water, as the mobile phase, was passed through the TEC at a known flow rate (1·0 ml/min) and the retained phenobarbitone eluted. The volume of water required to elute the phenobarbitone was thus estimated. With the TEC and dialyser repositioned in the system, this volume was not exceeded. The amount of packing material in the TEC was large enough to give a K′ for phenobarbitone of 7 ml. This was sufficient to obtain effective dialysis times and flow rates.

*Process times*

Like the system described in Part I, the process times were dependent on the dialyser size, volumes of connecting tubes, and pump tube flow rates. The time taken to aspirate sample into the dialyser was determined as in Part I and used identical pump and connecting tube sizes. However, in this procedure the sample was then held in the donor side of the dialyser block during trace enrichment and the TEC pump times have to be considered. The time taken for trace enrichment depends on both the K′ of the analyte and its concentration in the sample to be assayed. In this assay the time taken for trace enrichment was 2 min. This gave sufficient sensitivity for the detection of therapeutic concentrations of phenobarbitone at 0·05 AUFS on the UV detector. The flow rate of the recipient stream through the dialyser also depends on the dead volume of the system. If the dead volume is large, high flow rates are required to minimize both trace enrichment and wash cycle times. With the Gilson 302 pump, a flow rate of 2·5 ml/min was employed. This was sufficient to limit the wash cycle time to 1 min.

*Precision of the system*

Figure 4 shows the chromatograms obtained with the treated serum samples using the system described. The performance of the system was examined by estimating the within-run assay variance, with and without reference to the internal standard. 0·5 ml of stock phenobarbitone and 0·5 ml of stock hexobarbitone solution was diluted to 100 ml with pooled human serum. This provided a serum phenobarbitone concentration of 30 mg/l, which was within the recognized therapeutic range of 15–40 mg/l [3] for this drug. Thirty samples of the pooled supplemented serum were analysed through the dialysis, TEC system. The coefficient of variation with and without reference to hexobarbitone was 1·5% and 1·4% respectively. Carry-over between samples was determined by sampling a serum sample (supplemented with phenobarbitone and hexobarbitone), followed by a water sample. No carry-over could be detected. The procedure was found to be linear up to 100 mg/l of phenobarbitone in serum.

*Efficiency of the system*

The efficiency of the system was determined by substituting the dialysis/TEC components with a standard 20 µl loop on the 7010 Rheodyne valve and injecting known quantities of phenobarbitone and hexobarbitone onto the HPLC column. The peak areas obtained were compared with those obtained using the dialysis/TEC system described. The recovery of the analytes were calculated on the basis of the volume of the donor side of the dialyser and found to be 28 and 32% for phenobarbitone and hexobarbitone respectively.

### Discussion

The automatic sequential trace enrichment of dialysates (ASTED) suggests that this type of process for the automatic preparation of complex biological material prior to HPLC may offer some advantages over conventional procedures.

One of the principal problems encountered with assays of biological material using HPLC is the clean-up techniques that are required. Manually these are usually performed by protein precipitation [4] and liquid/liquid extractions [5]. Even the interfering compounds can affect the chromatography [6]. With dialysis/TEC sample preparation, the chromatograms appeared free from interfering peaks (figure 4). This resulted in an extended life of the TEC because it can be regenerated for many cycles (over 250 injections have been made with one TEC) and the life of the analytical column was prolonged. Furthermore, guard columns were found to be unnecessary, so efficiencies of small particle columns (<5 µm) were not reduced.

Although dialysis is not regarded as an efficient process analyte recoveries of greater than 50% from biological material should be possible with future modifications. This is because the system approaches exhaustive dialysis by holding the donor side in a stop–flow state and moving the recipient stream in a continuous mode across the membrane and into the TEC. However, exhaustive dialysis is unrealistic because the dialysis rate will decrease as the sample analyte concentration decreases and thus the time taken for analysis would be very long. In the case of phenobarbitone, total exhaustion of the analyte from the donor to the recipient stream is also impracticable because (1) the total volume of the

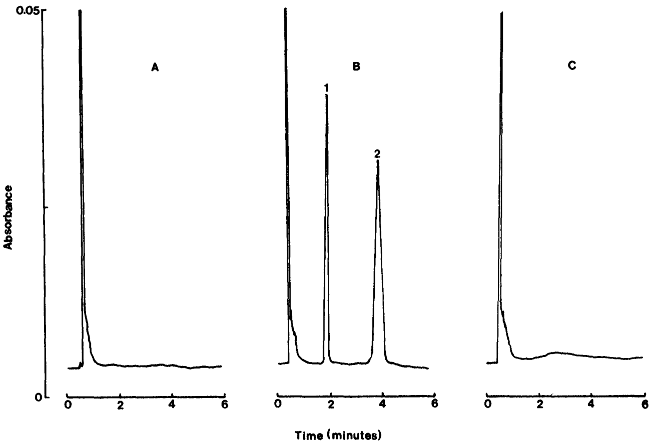

*Figure 4. Chromatograms of the following samples using the system described in the text: A, water sample; B, pooled serum supplemented with phenobarbitone (1) and hexobarbitone (2); C, same pooled serum before supplementing with drugs.*

recipient required would exceed the K′ of the analyte for the TEC; and (2) the time required would inhibit fast throughput of samples. In practice, this is not a problem since the recoveries of the analyte are sufficient to obtain adequate sensitivity.

The variance between runs was minimized by (*a*) constant dialysis sample volume; and (*b*) constant dialysis recipient volume. The Gilson 302 pump used for the trace enrichment was probably over the specification required for this procedure, since high back-pressures should not be experienced with the small quantities of trace enrichment material used. However, it was considered necessary to ascertain the potential of this system, so reliable and accurate pumping of the recipient stream through the dialyser into the TEC was essential. The TEC pump is positioned between the dialyser and the TEC to avoid rupturing the dialyser membrane.

This report describes a new approach to sample preparation prior to HPLC. Preliminary results using the system to estimate phenobarbitone in human serum demonstrate a promising potential for this technique. The procedure may also be applicable to a wide range of analytes in complex material since there are also a number of variables that can be altered within the system. For instance: the material used in the TEC, the nature of the recipient stream, the molecular weight cut off of the dialyser membrane, the size of the dialyser block and the process times may all be changed to suit different analyses.

### References

1. Lankelma, J. and Hoppe, H., *Journal of Chromatography*, **149** (1978), 587.
2. UK Patent Application 2 124370A.
3. Butler, T. C., in *Antiepileptic drugs: quantitative analysis and interpretation*, Eds. Pippenger, C. E., Penry, J. K. and Kutt, H. (Raven Press, New York, 1978), p. 268.
4. Kabra, P. M., Stafford, B. E. and Marton, L. J., *Clinical Chemistry*, **23** (1977), 1284.
5. Adams, R. F., Schmidt, G. J. and Vandemark, F. L., *Journal of Chromatography*, **145** (1978), 275.
6. Turnell, D. C., Trevor, S. C. and Cooper, J. D. H., *Annals of Clinical Biochemistry*, **20** (1983), 37.

### Acknowledgements

For both Parts I and II of this article we wish to thank: Mr R. W. Richardson for his continued support, Mr R. W. Fuller for advice and machining the TEC, and the following laboratory personnel for their expert technical assistance, Miss N. Shearsby, Mrs C. Fuller, Mr J. McIntosh and Mr M. Lewis, Gilson International for the loan of their HPLC pumps and Mr D. Hawkes of Anachem Ltd, for his encouragement.

**ABSTRACTING**

In keeping with our academic status *Journal of Automatic Chemistry incorporating The Journal of Clinical Laboratory Automation* is covered by the following abstractors:

Current Contents/Life Sciences (Institute for Scientific Information, Philadelphia, USA).

ISI/BIOMED (ISI, USA) Science Citation Index (ISI, USA).

Excerpta Medica (Excerpta Medica, Amsterdam, The Netherlands).

Analytical Abstracts (Royal Society of Chemistry, London).

Chemical Abstracts (American Chemical Society, Columbus, Ohio, USA).

